# Egyptian school children awareness and precautions in Covid19 pandemic: a cross sectional survey study

**DOI:** 10.1186/s42269-021-00495-0

**Published:** 2021-02-08

**Authors:** Manal A. Shehata, Ahmed Adel, Ayman F. Armaneous, Mohamed M. EL-Sonbaty, Mohamed Abdel Atti, Hazem Mohamed El-Hariri, Iman H. Kamel

**Affiliations:** 1 Department of Child Health, National Research Centre, El-Bohouth Street, Dokki, Cairo, 12622 Egypt; 2 Department of Community Medicine, National Research Centre (NRC), Cairo, Egypt

**Keywords:** Covid19, Egypt, School–children, Adolescents, Awareness

## Abstract

**Background:**

COVID-19 (Corona Virus Disease 2019) is showing a wide global spread, and urgent joint international efforts is required to the control of this pandemic, the awareness of people towards infectious viruses still the main factor to limit the widespread of disease. The aim of this study is to assess the level of awareness and attitude towards COVID-19 among a sample of Egyptian school children, using a web-based questionnaire.

**Results:**

A total of 708 participants were involved in this online survey study, representing different areas in Egypt, 378 males (53.4%), 330 females (46.5%); their age range between 6 and 18 years. Regarding the residence, 576 (81.4%) were from urban areas, the remaining 132 (18.6%) were from non-urban areas. Internet and media were more frequent used as a source of information in urban students. The knowledge level score of risk and prevention of the disease were significantly higher in urban students than in non-urban students. Healthy practice score ≥ 50 were significantly more frequent in urban students. Healthy practice score was non-significantly higher in urban students.

**Conclusions:**

Most of the study participants of school students are knowledgeable about basic information, and have cautious preventive practices towards COVID-19, denoting the efficacy of the public health efforts. However, the lower level of awareness in non-urbans, indicating a need to address alternative channels to communicate with these populations

## Background

The new coronavirus disease 2019 (COVID-19) has evolved into a pandemic, requiring persons around the world to attend to rapidly changing messages about public health and take urgent measures to control the spread of the virus (Wolf et al. 2020).

COVID-19 disease was identified in December 2019 in Wuhan, China. COVID-19 is highly contagious, the World Health Organization (WHO) declared it as a global health emergency on 30 January 2020 (Zhu et al. 2020), and as a pandemic in 11th of March 2020 (WHO 2020; Centers for Disease Control and Prevention 2019). The person-to-person transmission rate of SARS-CoV-2 has estimated to be 1.2–2.2% (Lu and Shi 2020). Since then, the infection has spread worldwide causing more than 15.6 million cases and 638 thousand deaths. Egypt Ministry of health stated the diagnoses of 90,413 confirmed Covid-19 cases, with 4480 reported deaths, in July 2020 (WHO 2020; The Center for Systems Science and Engineering 2020). Symptoms of COVID-19 disease include, fever, cough, fatigue, myalgia and headaches (Wang et al. 2020). Although some reports suggest initially that SARS-CoV-2 infection in children appears to be uncommon, but pediatric cases started to be reported first in china and then Italy and worldwide, but with less infection rate (Zhonghua Liu Xing Bing Xue Za Zhi 2020; Istituto Superiore di Sanità 2020).

Person to person transmission is thought to occur mainly via respiratory droplets infection can also occur if a person touches an infected surface and then touches his or her eyes, nose, or mouth. Droplets typically do not travel more than six feet (about two meters) (Van Soremalen et al. 2020). Negative attitudes and practices against new infectious diseases can exaggerate the epidemics which may finally result in pandemics (Alahdal et al. 2020). Awareness, Attitude and practice have been studied in many previous epidemics such as swine influenza (Shilpa et al. 2014), and Middle East Respiratory Syndrome (MERS) (Alkot et al. 2016). Public health measures include personal protective measures (hand hygiene, respiratory etiquette), environmental measures, physical distancing measures, and travel-related measures. Physical distancing measures apply to individuals (e.g. isolation of cases and quarantine of contacts) or to communities, specific segments of the population, or to the population as whole. These measures are used to combat the global pandemic of COVID-19 (Pan et al. 2020; WHO 2020).

Better awareness of the disease along with positive attitudes and practices towards it have shown to help contain the spread of the causative viruses. So, the aim of this study is to assess the level of awareness and attitude towards COVID-19 among a sample of Egyptian school children.

### Aim of the study

The aim of this study is to assess the level of awareness and attitude towards COVID-19 among a sample of Egyptian school children.

## Methods

A cross sectional survey study, using a web-based questionnaire, it was carried for 15 days, from the 1st till the 15th of July 2020. The study recruited Egyptian school children, their age between 6 and 18 years, who agreed to participate in the study. The survey was conducted in Arabic languages and took about 15 min to be completed by the student himself or with the aid of his parents.

The questionnaire was designed in accordance with previously published literature and the survey was pre-tested for validation designed by our team to collect data including, demographic information, and data measuring students’ awareness and attitude towards COVID 19 (Abdelhafiz et al. 2020).

All test data was collected through online model by google form. Communication between the researchers and the participants was conducted if needed. The questionnaire consisted of 3 main domains; the first domain focuses on sociodemographic information such as age, education level, residence and gender of the participants. While the second domain was about the students’ level of knowledge about the disease (30 items) which is subdivided into 3 subdomains (risk factors, clinical presentation and prevention precautions) each of them is 10 items. The third domain is about students’ practice and it is consisted of 10 items. The possible responses were “agree”, “disagree” and “don’t know”.

Approval for the study was obtained from The Medical Research Ethical Committee of the National Research Center (NRC) (Registration no. 20-095). All the participants provided online written informed consent to participate in the study.

### Statistical methods

The collected data was coded, tabulated, and statistically analyzed using IBM SPSS statistics (Statistical Package for Social Sciences) software version 22.0, IBM Corp., Chicago, USA, 2013 and Microsoft Office Excel 2007.

The answers of responders in each section were evaluated for being correct or false, hence we calculated the scores of each domain by counting the number of correctly answered question, then such number is divided by overall number of questions then multiplying by 100 to get the percentage of correctly answered questions (scores).

Descriptive statistics was done for quantitative data as mean ± SD (standard deviation) for quantitative data, while it was done for qualitative data as number and percentage. Inferential analyses were done for quantitative variables using independent t-test. In qualitative data, inferential analyses were done using Chi square test and Fisher’s Exact test for variables with small expected numbers. The level of significance was taken at *P* value < 0.050 is significant, otherwise is non-significant.

## Results

This study was conducted on 708 students represent different areas in Egypt 378 male (53.4%), 330 females (46.5%); their age ranged between 6 and 18 years. 342 (48.3%) of them in primary school and 147 (20.8%) in preparatory school, 219 (30.9) in secondary school. Their school types have different categories Governmental (250 Student, 35.3%), Experimental (294 Student, 41.5%), private (60 Student, 8.5%), international (104 Student, 14.7%). Among the studied 708 cases, 576 (81.4%) were from urban areas, the remaining 132 (18.6%) were from non-urban areas. As shown in Table 1 All participants claimed that they had heard about COVID-19 except 2 students 0.3%. Regarding sources of information about COVID-19, Internet, media and medical staff were more frequent in urban students but the differences were significant only in internet sources, while family and friends were non-significantly more frequent in non-urban students.

**Table 1:** Descriptive statistics among the studied cases and according to residence

Variables	All cases (*N* = 708)	Residence		*P* value
		Urban (*N* = 576)	Non-urban (*N* = 132)	
Age (years)				
6–9	212 (29.9%)	179 (31.1%)	33 (25.0%)	0.554^#^
9–12	161 (22.7%)	130 (22.6%)	31 (23.5%)	
12–15	132 (18.6%)	104 (18.1%)	28 (21.2%)	
15–18	203 (28.7%)	163 (28.3%)	40 (30.3%)	
Sex				
Male	378 (53.4%)	315 (54.7%)	63 (47.7%)	0.148^#^
Female	330 (46.6%)	261 (45.3%)	69 (52.3%)	
Education				
Primary	342 (48.3%)	280 (48.6%)	62 (47.0%)	0.916^#^
Preparatory	147 (20.8%)	118 (20.5%)	29 (22.0%)	
Secondary	219 (30.9%)	178 (30.9%)	41 (31.1%)	
COVID-19 infected student	19 (2.7%)	16 (2.8%)	3 (2.3%)	1.000^§^
COVID-19 infected relative	120 (16.9%)	92 (16.0%)	28 (21.2%)	0.148^#^
Source of information about COVID-19				
Internet	465 (65.7%)	394 (68.4%)	71 (53.8%)	**0.001**^#^*
Media	401 (56.6%)	334 (58.0%)	67 (50.8%)	0.131^#^
Family	390 (55.1%)	308 (53.5%)	82 (62.1%)	0.072^#^
Friends	134 (18.9%)	105 (18.2%)	29 (22.0%)	0.322^#^
Medical staff	125 (17.7%)	107 (18.6%)	18 (13.6%)	0.179^#^

Bold value indicate significance of the data

Data presented as frequency (percent)

^#^Chi square test

^§^Fishers Exact test

*Significant (< 0.050)

Description of knowledge level of studied cases and frequencies of correct answers are shown in Table 2. Correct responses regarding risk knowledge (no. 1, 2, 6, 7, 8, 10), and Risk score ≥ 50 were significantly more frequent in urban students. Risk score was significantly higher in urban students than in non-urban students.

**Table 2:** Knowledge (risk, clinical presentation and prevention) among the studied cases and according to residence

Variables	Correct responses rates All cases (*N* = 708)	Residence		*P* value
		Urban (*N* = 576)	Non-urban (*N* = 132)	
*Risk knowledge*				
1. Know the cause of COVID-19	650 (91.8%)	536 (93.1%)	114 (86.4%)	**0.011**^#^*
2. Transmission of disease by cough or sneezing	685 (96.8%)	564 (97.9%)	121 (91.7%)	0.001*^§^
3. Transmission via droplets during speaking	594 (83.9%)	487 (84.5%)	107 (81.1%)	0.325^**#**^
4. Transmission through contaminated surfaces	666 (94.1%)	546 (94.8%)	120 (90.9%)	0.089^**#**^
5. Virus survival for days in metallic surfaces	516 (72.9%)	423 (73.4%)	93 (70.5%)	0.487^**#**^
6. No trans-placental or perinatal transmission	143 (20.2%)	126 (21.9%)	17 (12.9%)	0.020*^#^
7. No transmission via Breast milk	182 (25.7%)	157 (27.3%)	25 (18.9%)	0.049*^#^
8. No transmission via cooked food	407 (57.5%)	350 (60.8%)	57 (43.2%)	< 0.001*^#^
9. Money and bags are source of infection	611 (86.3%)	497 (86.3%)	114 (86.4%)	0.981^**#**^
10. Covid19 is not transmitted from pets	269 (38.0%)	239 (41.5%)	30 (22.7%)	< 0.001*^#^
Risk score, Mean ± SD	66.7 ± 17.0	68.1 ± 16.4	60.5 ± 18.0	^ < 0.001*
Risk score grade				
< 50	65 (9.2%)	45 (7.8%)	20 (15.2%)	0.008*^#^
≥ 50	643 (90.8%)	531 (92.2%)	112 (84.8%)	
*Clinical presentation knowledge*				
1. Possibility of asymptomatic Covid 19 patient	618 (87.3%)	506 (87.8%)	112 (84.8%)	0.351^**#**^
2. Fever	654 (92.4%)	535 (92.9%)	119 (90.2%)	0.286^**#**^
3. Coughing	668 (94.4%)	544 (94.4%)	124 (93.9%)	0.821^**#**^
4. Sore throat	653 (92.2%)	534 (92.7%)	119 (90.2%)	0.322^**#**^
5. Body aches	609 (86.0%)	498 (86.5%)	111 (84.1%)	0.479^**#**^
6. Diarrhea, vomiting, abdominal pain	502 (70.9%)	398 (69.1%)	104 (78.8%)	0.027*^#^
7. Loss of taste or smell	561 (79.2%)	457 (79.3%)	104 (78.8%)	0.888^**#**^
8. Pneumonia	606 (85.6%)	496 (86.1%)	110 (83.3%)	0.412^**#**^
9. Some patients may get difficulty in breathing and need ICU admission	634 (89.5%)	523 (90.8%)	111 (84.1%)	**0.023**^**#**^
10. Presence of clinical symptoms of Covid-19 needs medical consultation	667 (94.2%)	552 (95.8%)	115 (87.1%)	< 0.001*^#^
Clinical presentation score, Mean ± SD	87.2 ± 18.5	87.6 ± 17.9	85.5 ± 20.8	^0.257
Clinical presentation score grade				
< 50	33 (4.7%)	26 (4.5%)	7 (5.3%)	0.698^**#**^
≥ 50	675 (95.3%)	550 (95.5%)	125 (94.7%)	
*Prevention knowledge*				
1. Repeated hand washing for 20 s	691 (97.6%)	566 (98.3%)	125 (94.7%)	0.025*^§^
2. Avoid touching the face with unclean hand	680 (96.0%)	559 (97.0%)	121 (91.7%)	0.004*^#^
3. Hand sterilization with alcohol	682 (96.3%)	563 (97.7%)	119 (90.2%)	< 0.001*^§^
4 Mask wearing outside home	676 (95.5%)	556 (96.5%)	120 (90.9%)	0.005*^#^
5. Keep social distance > 2 m	667 (94.2%)	550 (95.5%)	117 (88.6%)	0.002*^#^
6. Good home ventilation	631 (89.1%)	519 (90.1%)	112 (84.8%)	0.080^**#**^
7. Keep surfaces sterile with sanitizers	676 (95.5%)	556 (96.5%)	120 (90.9%)	0.005*^#^
8. Healthy food rich in vitamin C	604 (85.3%)	501 (87.0%)	103 (78.0%)	0.009*^#^
9. Exposure to the sun	616 (87.0%)	501 (87.0%)	115 (87.1%)	0.965^**#**^
10. Regular physical exercise	554 (78.2%)	455 (79.0%)	99 (75.0%)	0.316^**#**^
Prevention score, Mean ± SD	91.5 ± 15.8	92.5 ± 13.8	87.2 ± 22.4	^0.010*
Prevention score grade				
< 50	15 (2.1%)	8 (1.4%)	7 (5.3%)	0.011*^§^
≥ 50	693 (97.9%)	568 (98.6%)	125 (94.7%)	

Bold values indicate significance of the data

Data presented as frequency (percent) unless mentioned otherwise

^#^Chi square test

^§^Fishers Exact test

*Significant (< 0.050)

Clinical presentation knowledge score (the percentage of correctly answered questions in this domain) was non-significantly different between urban and non-urban students. However, correct responses for items (9,10) were significantly more frequent in urban students, while item no. 6 was significantly more frequent in non-urban students.

Prevention knowledge score (the percentage of correctly answered questions in this domain) was significantly higher in urban students, correct responses for items 1,2,3,4, 5,7, and prevention score ≥ 50 were significantly more frequent in urban students.

Healthy practice among the studied cases and according to residence is demonstrated in Table3. Correct responses for items no. 1, 6, 7, 9, 10 and healthy practice score ≥ 50 were significantly more frequent in urban students. Healthy practice score was non-significantly higher in urban students in urban students.

**Table 3:** Healthy practice among the studied cases and according to residence

Variables	Correct responses rates All cases (*N* = 708)	Residence		*P* value
		Urban (*N* = 576)	Non-urban (*N* = 132)	
1. Hand washing directly when coming from outside most of the times	665 (93.9%)	550 (95.5%)	115 (87.1%)	< 0.001*^#^
2. Hand washing before and after eating most of the times	611 (86.3%)	496 (86.1%)	115 (87.1%)	0.761^#^
3. Covering nose and mouth while coughing or sneezing	617 (87.1%)	505 (87.7%)	112 (84.8%)	0.382^#^
4. Wearing mask outside home	480 (67.8%)	399 (69.3%)	81 (61.4%)	0.079^#^
5. Keeping social distance for most of the time	555 (78.4%)	456 (79.2%)	99 (75.0%)	0.294^#^
6. Greeting others without handshaking or kisses	647 (91.4%)	534 (92.7%)	113 (85.6%)	0.009*^#^
7. Avoid using tools of others	606 (85.6%)	503 (87.3%)	103 (78.0%)	0.006*^#^
8. Eating plenty of vegetables and fruits daily	513 (72.5%)	419 (72.7%)	94 (71.2%)	0.722^#^
9. Regular exposure to the sun	321 (45.3%)	249 (43.2%)	72 (54.5%)	0.018*^#^
10. Follow the instruction of staying at home	609 (86.0%)	506 (87.8%)	103 (78.0%)	0.003*^#^
Practice score, Mean ± SD	79.4 ± 20.9	80.2 ± 19.5	76.3 ± 26.1	^0.111
Practice score grade				
< 50	53 (7.5%)	36 (6.3%)	17 (12.9%)	0.009*^#^
≥ 50	655 (92.5%)	540 (93.8%)	115 (87.1%)	

Bold values indicate significance of the data

Data presented as frequency (percent) unless mentioned otherwise

^#^Chi square test

^§^Fishers Exact test

*Significant (< 0.050)

## Discussion

COVID-19, is showing dramatic growth all over the world Since the middle of March in 2020. The principal causes of this wide spread of the disease include unawareness of the novel coronavirus, deficient capacity in detection, and delayed policy-making and implementing of appropriate prevention and control means through the early stages of the epidemic (Marzia et al. 2020). The disease now is in the state of pandemic, with no specific medication and a prolonged vaccines development cycle (Paul and Katherine 2020), efficient intervention procedures will play an important role, and all the world should cooperate to control the spread of the virus. Accordingly, it is necessary to explore the knowledge and practices of the society towards COVID-19 to initiate beneficial strategies (Zhao et al. 2020).

In this study, we investigated the awareness and precautions practices of school students from Egypt, towards the COVID-19 pandemic. Considering that, exploration of socioeconomic characteristics of the participant is required, to be utilized in guiding the mapping of awareness campaigns and to find out whether people’s knowledge differed according to certain characteristics of the target populations, this study is shedding light on the difference of students' awareness according to their residence whether urban or non-urban.

The majority of this study group were from urban residence (81.4%), that could be attributed to the weak internet services in non-urban areas, as this study is web-based. Moreover, internet sources of information about COVID-19, were used more frequent in urban students with a significant difference in comparison to non-urban. Whereas, the main source of information for non-urban students was their families. This finding is strongly supported by similar report regarding Egyptian adults in which the principal source of covid-19 information was found to be the internet and social media (Abdelhafiz et al. 2020).

The majority of the participants in this survey have overall good general knowledge regarding the risk of disease transmission, the common clinical presentations and the preventive measures. However, there were significant differences between urban and non-urban groups regarding several items in each category. Besides the significant higher prevention and risk knowledge scores in urban group. Nevertheless, less knowledge was found regarding some items such as the virus survival on metallic surfaces and presence of abdominal symptoms (vomiting and diarrhea) as a part of the clinical manifestations. We also observed some false beliefs concerning the disease transmission from pets and via cooked food.

In close agreement with this findings, a similar cross-sectional survey study conducted on Egyptian adults reported a good knowledge about covid-19 and a positive attitude regarding prevention practices. In addition, they found a lower levels of awareness associated with the rural residence and low socio-economic standard (Abdelhafiz et al. 2020). Similarly, Hager et al. (2020) assessed the awareness of Egyptians and Nigerians adults towards COVID-19, they reported a satisfactory level of awareness, as well in more than 60% of the participants.

On another aspect, hand hygiene and wearing of face masks were evidenced to be efficient in preventing the spread of the virus throughout the epidemic of severe acute respiratory syndrome (Jefferson et al. 2009). A Chinese study was conducted on primary school children to assess the status of hand hygiene and wearing of face mask amongst them. 42.05% of the primary school children performed a satisfactory practice of hand-washing, whereas 51.60% had a proper practice of mask wearing (Chen et al. 2020).

The prevention practices of the students in our study were very cautious, the mean practice score was 79.4 ± 20.9, with (92.5%) of respondents have ≥ 50 degrees, (93.9%) practiced hand washing directly when coming from outside, and (86%) followed the instructions of staying at home, while 67.8% reported their adherence to wearing face-mask when going outside home.

These precautions practices could be attributed to the participants’ good awareness of the disease regarding the infectivity of the virus. As several studies have proved the importance of awareness of people to control infection throughout epidemics and pandemics (Alahdal et al. 2020; Almutairi et al. 2015). Improving the awareness towards pandemics, require governmental efforts to increase awareness among society, using diverse communications routes. In spite of the marked government efforts and the great media role in the health education about COVID-19 in our country still, societies with lower socioeconomic levels may need alternate channels for communication (Hoda 2016).

### Study limitations

This study is web based so the poor internet services especially in rural areas of Egypt is considered a main limitation, in addition, young students may need the help of their parents, so people from poor and less educational background, face difficulty in participation, and their awareness level may differ from our findings. Therefore, the results of this study cannot be generalized to the whole Egyptian populations, however, the study provides an initial overview of the awareness level of school children about Covid-19, and indicates the importance of policies that target at risk populations, it also emphasizes the need for larger scale studies with more suitable routes of communications in urban areas.

## Conclusions

Most of the study participants of school students are knowledgeable of basic information, and have cautious preventive practices towards COVID-19, denoting the efficacy of the public health efforts. However, the study findings revealed lower level of awareness in non-urban areas, indicating a need to intensify the awareness program and address alternative channels to communicate with these populations. School Children especially in rural areas need suitable means to deliver information, it can be represented in the form of children friendly booklets, boosters, role modeling plays, demonstrative cartoon movies, and other routes preferred to children, so that they are improving awareness of data regarding COVID-19 to overcome gap of Knowledge.

## Data Availability

Data available by request from child health department NRC Egypt. Authors may be contacted at dr.ahmedadell@yahoo.com.

## Abbreviations

COVID-19: Corona Virus Disease 2019
WHO: World Health Organization
MERS: Middle East Respiratory Syndrome
SPSS: Statistical Package for Social Sciences
